# Effects of Dietary Supplementation of *Lactobacillus delbrueckii* on Gut Microbiome and Intestinal Morphology in Weaned Piglets

**DOI:** 10.3389/fvets.2021.692389

**Published:** 2021-08-19

**Authors:** Xiao-Long Wang, Zhu-Ying Liu, Ying-Hui Li, Ling-Yuan Yang, Jie Yin, Jian-Hua He, De-Xing Hou, Ya-Li Liu, Xing-Guo Huang

**Affiliations:** ^1^College of Animal Science and Technology, Hunan Agricultural University, Changsha, China; ^2^College of Animal Science and Technology, Hunan Biological and Electromechanical Polytechnic, Changsha, China; ^3^The United Graduate School of Agricultural Science, Kagoshima University, Kagoshima, Japan; ^4^Hunan Pufeike Biotechnology Company, Changsha, China

**Keywords:** *Lactobacillus delbrueckii*, mucosal morphology, gut microbes, short chain fatty acids, weaned piglets

## Abstract

*Lactobacillus delbrueckii* is a Gram-positive bacterium mostly used in the dairy industry for yogurt and cheese. The present study was designed to evaluate the effects of *Lactobacillus delbrueckii* on serum biochemical parameters, intestinal morphology, and performance by supplementing at a dietary level of 0.1% in diets for weaned piglets. Eighty healthy weaned piglets (initial body weight: 7.56 ± 0.2 kg) were randomly divided into two feeding groups with four replicates in each group (n = 10 animals per replicate); piglets were fed with basal diet (CON) or basal diet containing 0.1% *Lactobacillus delbrueckii* (LAC). The results showed that dietary supplementation of *Lactobacillus delbrueckii* improved growth performance and increased serum HDL and insulin levels in piglets on the 28th day of the experimental time (*p* < 0.05). The gut microbe analysis revealed that *Lactobacillus delbrueckii* significantly decreased the relative abundance of the phyla Bacteroidetes, but increased the relative abundance of the phyla Firmicutes. The *Lactobacillus delbrueckii* also significantly increased the relative abundance of *Bifidobacterium* and *Lactobacillus* at the genus level of the bacterial community in the ileum, but decreased the relative abundance of unclassified *Clostridiales*. Moreover, *Lactobacillus delbrueckii* improved mucosal morphology by obtaining higher intestinal villus height (*p* < 0.05), significantly increasing the concentrations of butyrate, isobutyric acid, and isovaleric acid in colonic chyme of piglets, but decreasing the intestinal pH at the duodenum and ileum on the 28th day of the experimental time. In conclusion, dietary supplementation of *Lactobacillus delbrueckii* in the diet of weaned piglets can improve intestinal morphology and modulate the microbiota community to promote growth performance.

## Introduction

Weaning is one of the most stressful stages in pig production. It could cause intestinal and immune system dysfunctions, resulting in compromised growth performance and inferior disease resistance of piglets (1, 2). Also, it could cause drastic changes in intestinal morphology, such as shortening of the villus, elongation of the crypt (3, 4), and even chronic impairment of the mucosal barrier function (5).

Probiotics are living non-pathogenic bacteria that have beneficial effects on the host animals by modulating their intestinal microbial structure, and they are used as feed additives in animal production (6). The effects of some probiotic strains on improvements of intestinal microbial balance were observed in piglets and poultry (7, 8). The gastrointestinal system has multiple functions such as maintaining humoral balance, secreting digestive enzymes, immunoglobulins, and other components, and it is also a barrier for the host to resist harmful pathogens and antigens. Many studies have found that *Lactobacillus* could improve intestinal morphology and gut microbiota to prevent intestinal barrier dysfunction (9, 10). Thus, *Lactobacillus* has been used to promote growth and intestinal integrity in animals. However, the mechanisms of how probiotic *Lactobacillus* worked were not fully elucidated.

In our previous study, oral administration of *Lactobacillus delbrueckii* to suckling piglets improved the immune response, intestinal morphology, barrier function, and growth (11). *Lactobacillus delbrueckii* is a Gram-positive bacterium mostly used in the dairy industry for yogurt and cheese, exerting beneficial probiotic roles (12). In addition, our previous results showed that dietary supplementation of 0.1% *Lactobacillus delbrueckii* improved the growth performance of fattening pigs (13). The current study was designed to evaluate the effects of *Lactobacillus delbrueckii* on serum biochemical parameters, intestinal morphology, and gut microbial construction by supplementing with *Lactobacillus delbrueckii* at a dietary level of 0.1% of the diets for the weaned piglets.

## Materials and Methods

### Animals, Diets, and Experimental Design

Eighty healthy weaned piglets (Landrace × Yorkshire × Duroc) of mixed sex were randomly divided into two groups with four replicates (pens), each pen with 10 pigs (male and female half). The experiment started when the piglets reached 28 days of age after weaning. The control (CON) group was fed a basal diet (without antibiotic); the *Lactobacillus delbrueckii* (LAC) group was fed a basal diet containing 0.1% *L. delhrueckii* (1.0 × 10^10^CFU/g). Feed and water were provided *ad libitum* throughout the experiment period. The experimental lasted 28 days and was divided in two phases (phase I from d 1 to 14, and phase II from d 15 to 28). The basal diets were formulated based on the nutrient requirements of swine (NRC, 2012) (Table 1).

**Table 1:** The composition of the basal diet.

**Item**	**Content**	**Nutrient levels^a^**	**Content**
**Ingredients (%)**			
Corn	62.00	DE(MJ/kg)	14.11
Extruded soybean	10.00	Crude protein (%)	18.47
Soybean meal	14.00	CEE (%)	4.40
Low protein whey powder	5.00	Lysine (%)	1.30
Fermented soybean	3.00	Methionine (%)	0.39
Fish meal	2.50	Threonine (%)	0.80
Calcium phosphate	1.40	Met+Cys (%)	0.70
Limestone	0.40	Ca (%)	0.70
Choline chloride	0.10	Total phosphorus (%)	0.63
NaCl	0.20	Available phosphorus (%)	0.44
Lysine	0.42		
DL-Methionine	0.08		
Threonine	0.10		
Premix^b^	0.80		
Total	100.00		

*DE, digestible energy; CEE, crude fat ether extract; Met, DL-methionine; Cys, cysteine; Ca, calcium*.

a *Based on the Nutrient Requirements of swine (NRC, 2012)*.

b *Supplied, per kilogram of diet: Cu, 100 mg; Fe, 90 mg; Mn, 40 mg; Zn, 80 mg; Se, 0.3 mg; I, 0.6 mg; vitamin A, 9,000 IU; cholecalciferol, 2,800 IU; vitamin E, 22 IU; thiamine, 3 mg; riboflavin, 7.0 mg; pyridoxine, 4.0 mg; cobalamin, 30 μg; niacin, 30 mg; pantothenic acid, 10 mg; folic acid, 0.32 mg; biotin, 0.2 mg*.

### Preparation of *Lactobacillus delhrueckii*

The *Lactobacillus delhrueckii* CCTCC M207096 was obtained from the microbiology laboratory of the College of Animal Science and Technology, Hunan Agricultural University, and prepared as microcapsule granules (1.0 × 10^10^ CFU/g) by the Hunan Pufeike Biotechnology Company. The *Lactobacillus delhrueckii* was enveloped by the binder, microcrystalline cellulose, and other excipients, followed by water granulation, shot blasting and drying, and finally prepared into particles. The *Lactobacillus delhrueckii* was heated during the processing. The number of *Lactobacillus delhrueckii* (1.0 × 10^10^ CFU/g) was measured by viable bacteria.

### Sample Collection and Preparation

Feed intake per pen was recorded daily. The growth rate and feed conversion ratio (feed/gain) for two different experimental periods were calculated. At the 14th and 28th day of the experiment, four piglets (one per replicate) with medium body weights in each pen were selected. The blood samples for each selected piglet were collected by venipuncture into 15-ml tubes and centrifuged at 3,000 × *g* for 10 min at 4°C. The supernatants (serum) were collected for serum biochemical analyses. After blood collection, the piglets were slaughtered by exsanguination after electrical stunning.

About 2 g of digesta were taken from the middle of the ileum, cecum, and colon, respectively, after the weaned piglets were euthanized for analysis of microbial diversity. Feces (~1 g) were collected for short-chain fatty acid (SCFA) determination. Digesta and feces samples were separately stored at −80°C. The small intestinal sections, including the duodenum from the pyloric sphincter to the duodenojejunal bend, the jejunum, which ended at the attachment of the plica ileocecalis, and the ileal, which ended at the ileocecal opening (14), were quickly removed and divided into three parts. A segment (2 cm) of each intestinal section was collected for mucosal morphology analysis.

### Serum Biochemical Parameters

The concentrations of total protein (TP), triglycerides (TG), total cholesterol (TC), high-density lipoprotein (HDL), low-density lipoprotein (LDL), and the activities of alanine aminotransferase (ALT) and aspartate aminotransferase (AST) in the serum of the piglets were analyzed using the BS-200 automatic blood biochemical analyzer (Mindray, Shengzhen, China).

### Measurement of Intestinal Mucosal Morphology

The 2-cm intestinal tissue samples were stained with hematoxylin and eosin (HE) as described previously (11). Villus height (from the villi tip to the villus–crypt joint) and crypt depth (from the villus–crypt joint to the base of the crypt) were measured under an Olympus Van-Ox S microscope (Opelco, Washington, DC, USA) using an image analysis software (Image-Pro, Media Cybernetics, Inc., Silver Springs, MD, USA). Ten sections were taken from each slice for the measurements. The villus height/crypt depth (V/C) value was calculated.

### Measurement of Intestinal pH

Immediately after slaughtering, the pH values of the basal glandular areas of the small intestine, cecum, colon, and rectum were measured *in situ*. The pH values of the small intestine were measured at fixed points, which were at 1 cm distal from the pylorus, and at 1/16, 1/8, 1/4, 1/2, 3/4, and 4/4 of the length of the small intestinal sections. All pH measurements were tested by inserting the pH probe into the cavities through a small incision on the gut wall (portable Sentron pH meter type Argus with Lancefet probe, The Netherlands, Sentron Europe B.V., Roden,).

### DNA Extraction and Cecal Microbiota Analysis of Fecal Samples

Total microbial genomic DNA samples were extracted using the QIAamp DNA Stool Mini Kit (QIAGEN, Inc., Netherlands) and stored at −20°C prior to further analysis. The concentration and quality of the extracted DNAs were determined by the NanoDrop ND-1000 spectrophotometer (Thermo Fisher Scientific, Waltham, MA, USA) and agarose gel electrophoresis, respectively. The V3–V4 hypervariable region of the bacterial 16S rRNA gene was amplified by PCR with the forward primer 338F: 5′-ACTCCTACGGGAGGCAGCAG-3′ and the reverse primer 806R: 5′-GGAC- TACHVGGGTWTCTAAT-3′. Sample-specific 7-bp barcodes were incorporated into the primers for multiplex sequencing. The PCR components contained 5 μl of Q5 reaction buffer (5 ×), 5 μl of Q5 high-fidelity GC buffer (5 ×), 0.25 μl of Q5 high-fidelity DNA polymerase (5 U/μl), 2 μl (2.5 mM) of dNTPs, 1 μl (10 uM) of each forward and reverse primer, 2 μl of DNA template, and 8.75 μl of ddH_2_O. Thermal cycling consisted of initial denaturation at 98°C for 2 min, followed by 25 cycles consisting of denaturation at 98°C for 15 s, annealing at 55°C for 30 s, and extension at 72°C for 30 s, with a final extension of 5 min at 72°C. PCR amplicons were purified with Agencourt AMPure Beads (Beckman Coulter, Indianapolis, IN, USA) and quantified using the PicoGreen dsDNA Assay Kit (Invitrogen, Carlsbad, CA, USA). After the individual quantification step, amplicons were pooled in equal amounts, and paired-end 2′ 300-bp sequencing was performed using the Illlumina MiSeq platform with MiSeq Reagent Kit v3 at the Shanghai Personal Biotechnology Co., Ltd. (Shanghai, China) (15, 16). The OTUs, alpha diversity, beta diversity analysis, and the microbiota structure analysis were done according to previously described procedures (17). OTU taxonomy was assigned by the Greengene database. For α-diversity analysis, Chao 1 index was calculated by Mothur, and the Shannon index was calculated by the R package “vegan” to estimate the bacterial community richness within each sample. β-diversity was assessed by MANOVA and principal coordinate analysis (PCoA).

### Short-Chain Fatty Acid Quantification

Gas chromatography (Agilent 7890 A, Agilent Technologies, Santa Clara, CA, USA) was used to determine the concentrations of acetate, propionate, butyrate, i-butyrate, i-valerate, and valeric acid in colon contents according to the procedures described previously (18). One microliter of sample was injected into a 7890 Agilent gas chromatograph. Nitroterephthalic acid-modified polyethylene glycol column (DB-FFAP) was used for the gas chromatograph. The column temperature was operated at 250 to 280°C, and the carrier gas was 0.8 ml/min of high-purity N_2_. The minimum detectable thresholds for all volatile fatty acids (VFA) were 0.1 mmol/L.

### Statistical Analysis

Data in tables and figures were expressed as means ± SEM and means ± SD, respectively, and data were analyzed by *T*-test using SPSS 20.0 (SPSS, Inc., Chicago, IL, USA). Significance was defined as a *p*-value < 0.05%. The statistical analysis used in the assessment of the microbial community structure of α-diversity and OTU counts were determined by *T*-test using SPSS 20.0 as well.

## Results

### Growth Performance

The results of Table 2 shows the growth performance in the weaned piglets. For the phase I (1 to 14 d), phase II (15 to 28 d), and the entire experimental period, piglets in the LAC group had higher ADG and BW when compared with the CON group (*p* < 0.05). There were no differences (*p* > 0.05) in ADFI and FCR between the LAC group and the CON group at phase I and the phase II, but the LAC group had higher ADFI and lower FCR values than the CON group throughout the entire experimental period (*p* < 0.05).

**Table 2:** Effects of *Lactobacillus delbrueckii* on growth performance in weaned piglets.

**Item**	**Time**	**CON**	**LAC**	**SEM**	***P*-value**
BW (kg)	1 d	7.53	7.6	0.10	0.508
	14 d	11.46^b^	11.91^a^	0.18	0.044
	28 d	18.68^b^	19.49^a^	0.29	0.032
ADFI (g)	1–14 d	438.33	453.2	9.80	0.18
	15–28 d	854.61	881.78	15.29	0.126
	1–28 d	646.47^b^	667.48^a^	8.18	0.042
ADG (g)	1–14 d	280.71^b^	308.42^a^	9.53	0.027
	15–28 d	516^b^	541.2^a^	9.43	0.037
	1–28 d	398.07^b^	424.88^a^	8.84	0.028
FCR	1–14 d	1.56	1.47	0.06	0.161
	15–28 d	1.66	1.63	0.04	0.474
	1–28 d	1.62^b^	1.57^a^	0.001	0.048

*CON, basal diet; LAC, basal diet group with 0.1% Lactobacillus delbrueckii; BW, body weight; ADG, average daily body weight gain; ADFI, average daily feed intake; FCR, feed/gain ratio. a, b within a row, values with different superscripts mean different significantly (p < 0.05). Each mean represents four replicates*.

### Serum Biochemical Parameters

The results of serum biochemical parameters are shown in Table 3. Although no significant differences on the serum concentrations of AST, LDL, TC, and TG were observed among groups throughout the entire experimental period (*p* > 0.05), the ALT concentration of piglets in the LAC group was lower than that in the CON group on the 28th day (*p* < 0.05). The TP concentration of piglets in the LAC group was higher than that in the CON group on the 14th day (*p* < 0.05). The HDL concentration of piglets in the LAC group was higher than that in the CON group on the 28th day (*p* < 0.05).

**Table 3:** Effects of *Lactobacillus delbrueckii* on serum biochemistry parameters in the weaned piglets.

**Item**	**Time**	**CON**	**LAC**	**SEM**	***P*-value**
TP(g/L)	14 days	49.24^b^	59.21^a^	2.07	<0.001
	28 days	46.51	48.91	1.77	0.223
TG (mmol/L)	14 days	0.33	0.44	0.06	0.128
	28 days	0.42	0.49	0.11	0.545
TC (mmol/L)	14 days	2.24	2.66	0.27	0.172
	28 days	2.29	2.03	0.23	0.294
HDL (mmol/L)	14 days	0.87	0.97	0.13	0.473
	28 days	0.81^b^	1.14^a^	0.08	<0.001
LDL (mmol/L)	14 days	1.00	1.24	0.18	0.242
	28 days	0.95	0.83	0.11	0.295
ALT(u/L)	14 days	78.63	101.04	15.03	0.186
	28 days	149.15^a^	109.67^b^	15.38	<0.05
AST(u/L)	14 days	63.58	73.33	10.43	0.386
	28 days	83.92	56.07	14.45	0.102

*CON, basal diet; LAC, basal diet group with 0.1% Lactobacillus delbrueckii; TP, total protein; TG, triglycerides; TC, total cholesterol; HDL, high density lipoprotein; LDL, low density lipoprotein; ALT, alanine aminotransferase; AST, aspartate aminotransferase. Different superscript lowercase letters within each group mean different significantly (p < 0.05). Each mean represents four replicates*.

### Intestinal Mucosal Morphology

**Figure 1:**
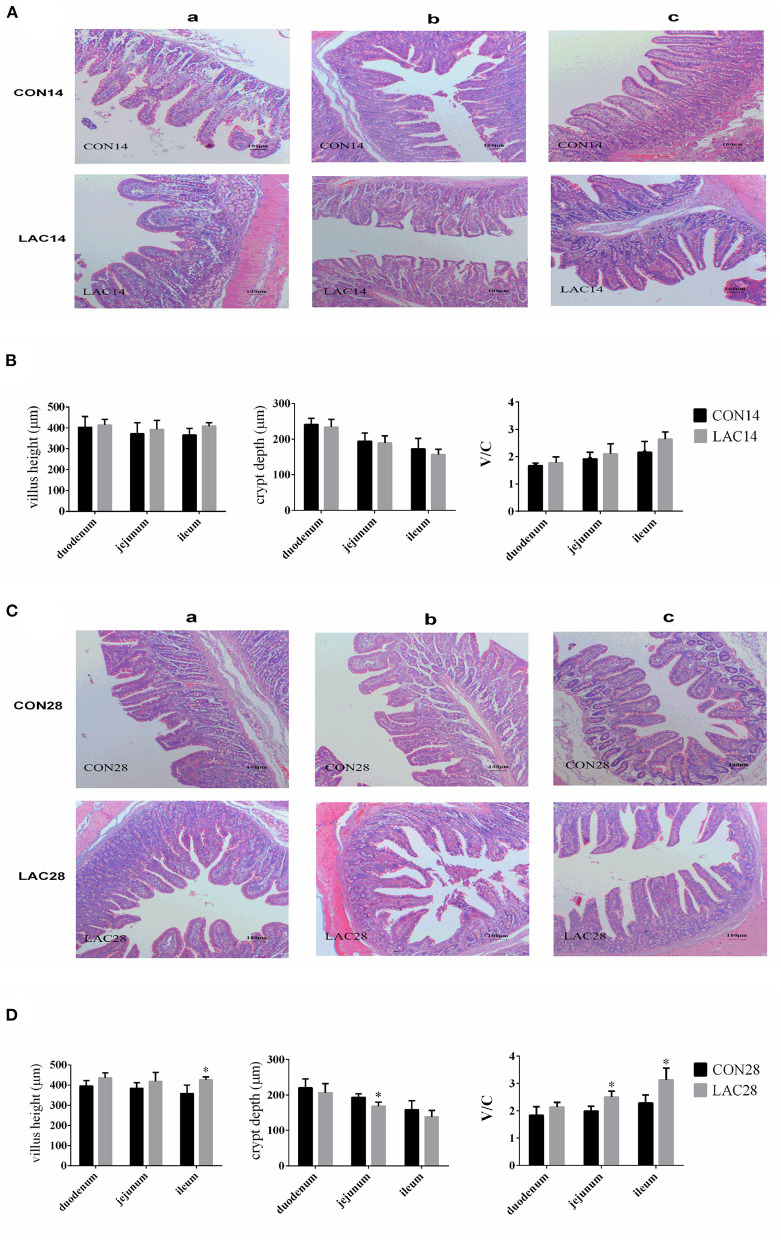
Effects of *Lactobacillus delbrueckii* on the intestinal mucosal morphology of the weaned piglets. **(A)** Intestinal (a: duodenum; b: jejunum; c: ileum) mucosal morphology was observed (40 ×) in weaned piglets on the 14th day. **(B)** The villus lengths, the crypt depths, and the V/C of the intestinal sections on the 14th day. **(C)** Intestinal (a: duodenum; b: jejunum; c: ileum) mucosal morphology was observed (40 ×) in weaned piglets on the 28th day. **(D)** The villus lengths, the crypt depth, and the V/C of the intestinal sections on the 28th day. *N* = 4. Label (*) mean significant difference between the CON group and the LAC group (*p* < 0.05). CON, basal diet; LAC, basal diet with 0.1% *Lactobacillus delbrueckii*.

The results of Figure 1 shows the morphologies of the intestinal mucosa in the duodenum, jejunum, and ileum. The villus height and V/C in the duodenum of the LAC group were not significantly affected throughout the entire experimental period (*p* > 0.05). The villus height, crypt depth, and V/C in the jejunum of the LAC group were not significantly affected on the 14th day (*p* > 0.05), but the crypt depth was lower, and the V/C was higher in the LAC group on the 28th day (*p* < 0.05). The villus height, crypt depth, and V/C in the ileum of the LAC group were not significantly affected on the 14th day, but the villus height and the V/C were significantly affected on the 28th day (*p* < 0.05).

### Intestinal pH

**Figure 2:**
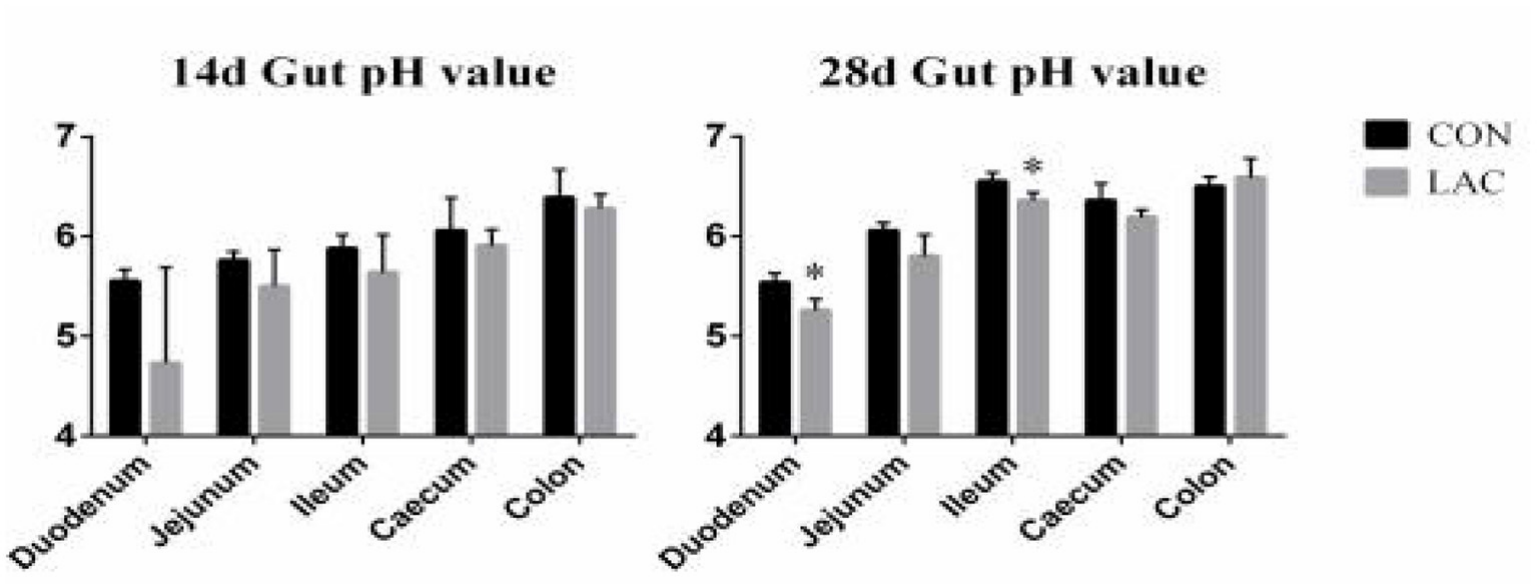
Effects of *Lactobacillus delbrueckii* on intestinal pH in weaned piglets. *N* = 4. Label (*) mean significant difference between the CON group and the LAC group (*p* < 0.05). CON, basal diet; LAC, basal diet with 0.1% *Lactobacillus delbrueckii*.

Figure 2 shows the pH values of the duodenum, jejunum, ileum, cecum, and colon. There were no significant differences in the intestinal pH between the two groups on the 14th day (*p* > 0.05), but the LAC group had lower pH values in the duodenum and ileum compared with the CON group on the 28th day (*p* < 0.05).

### The Gut Microbiota Composition

**Figure 3:**
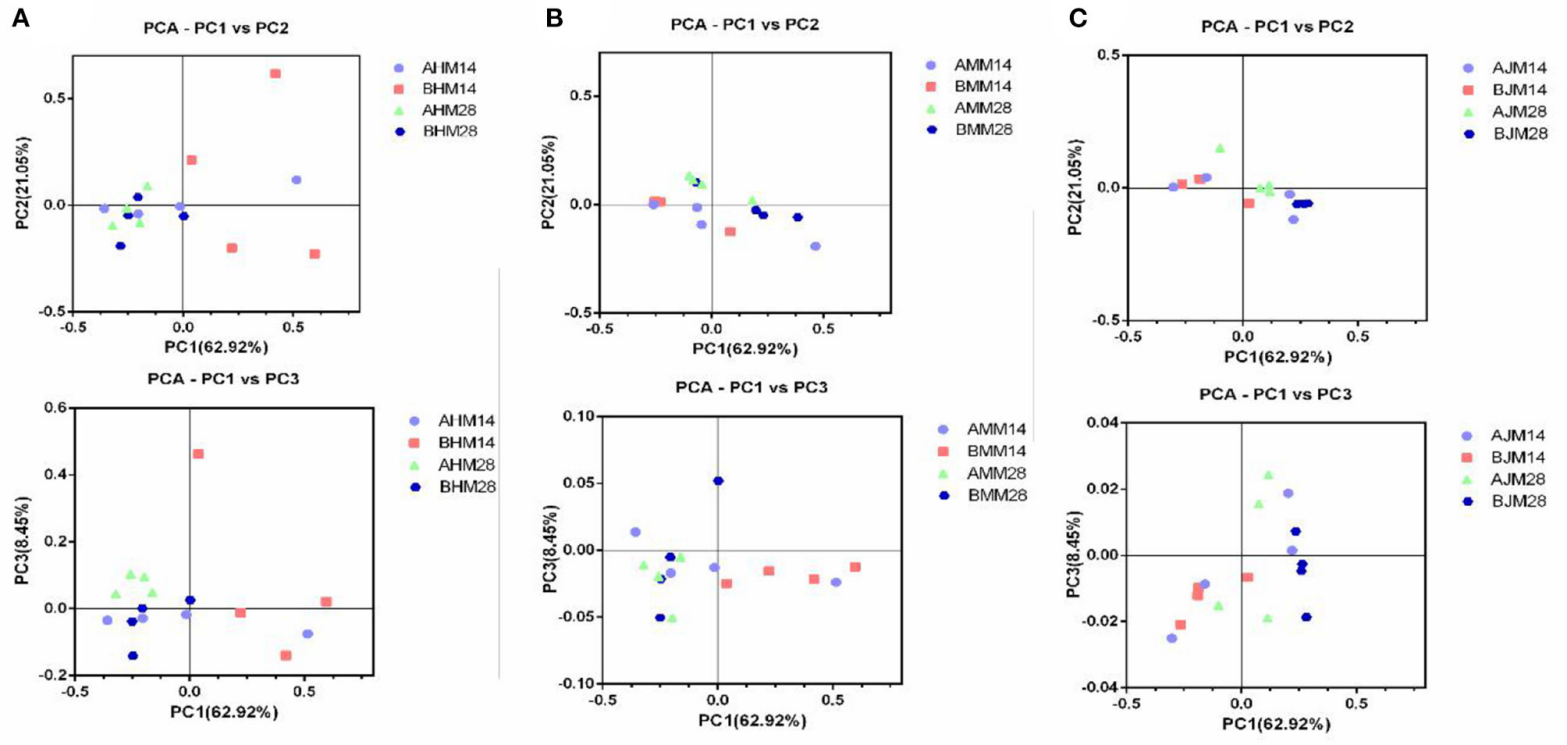
Effects of *Lactobacillus delbrueckii* on the β-diversity of the weaned piglets. **(A)** Microbial community β-diversity (unweighted Unifrac, *p* > 0.05) in the ileum of the piglets at 14 and 28 days, which was demonstrated using principal coordinates analysis (PCoA) of the unweighted Unifrac distance matrices. **(B)** Microbial community β-diversity (unweighted Unifrac, *p* > 0.05) in the cecum of the piglets at 14 and 28 days, which was demonstrated using principal coordinates analysis (PCoA) of the unweighted Unifrac distance matrices. **(C)** Microbial community β-diversity (unweighted Unifrac, *p* > 0.05) in the colon of the piglets at 14 and 28 days, which was demonstrated using principal coordinates analysis (PCoA) of the unweighted Unifrac distance matrices. Each dot represented one sample, and each group was denoted by a different color and shape. CON, basal diet; LAC, basal diet with 0.1% *Lactobacillus delbrueckii*.

To assess whether the gut microbiota was influenced by *Lactobacillus delhrueckii*, we performed α-diversity analyses on the digesta of the ileum, cecum, and colon. The results show that the dietary supplementation of *Lactobacillus delhrueckii* did have effects on Chao 1 and Shannon of the alpha diversity indexes of the microbial communities in the ileum, cecum, and colon (Table 4). At 14 days, piglets in the LAC group exhibited higher Chao 1 in the ileum, cecum, and colon (*p* < 0.05) when compared with piglets in the CON group. At 28 days, piglets in the LAC group exhibited higher Shannon indexes in the ileum (*p* < 0.05). Additionally, piglets in the LAC group exhibited higher Chao 1 in the cecum (*p* < 0.05) and higher Shannon indexes in the colon compared with the CON group (*p* < 0.05). However, β-diversity was not significantly different between the groups in the ileum, cecum, and colon (p>0.05), as shown in Figures 3A–C.

**Table 4:** Effects of *Lactobacillus delbrueckii* on the α-diversity of the microbiota in the weaned piglets.

**Gut**	**Item**	**Time**	**CON**	**LAC**	**SEM**	***P*-value**
Ileum	Chao1	14 days	306.45^b^	361.02^a^	16.92	<0.05
		28 days	899.99	1054.32	85.52	0.102
	Shannon	14 days	4.11	4.39	0.85	0.128
		28 days	5.39^b^	5.89^a^	0.19	<0.05
Cecum	Chao1	14 days	1060.74^b^	1302.69^a^	70.65	<0.05
		28 days	2355.74^b^	2864.23^a^	124.58	<0.05
	Shannon	14 days	6.11	7.19	0.93	0.275
		28 days	7.91	7.65	0.68	0.72
Colon	Chao1	14 days	1315.54^b^	1751.13^a^	114.84	<0.05
		28 days	3396.6	3739.3	289.58	0.052
	Shannon	14 days	6.11	7.19	0.93	0.304
		28 days	7.89^b^	8.99^a^	0.37	<0.05

*CON, basal diet; LAC, basal diet group with 0.1% Lactobacillus delbrueckii. Taxa richness assessed by α-diversity analyses using Chao1 and Shannon methods on ileum, cecum, and colon. Different superscript lowercase letters within each group mean different significantly (p < 0.05). Each mean represents four replicates*.

**Figure 4:**
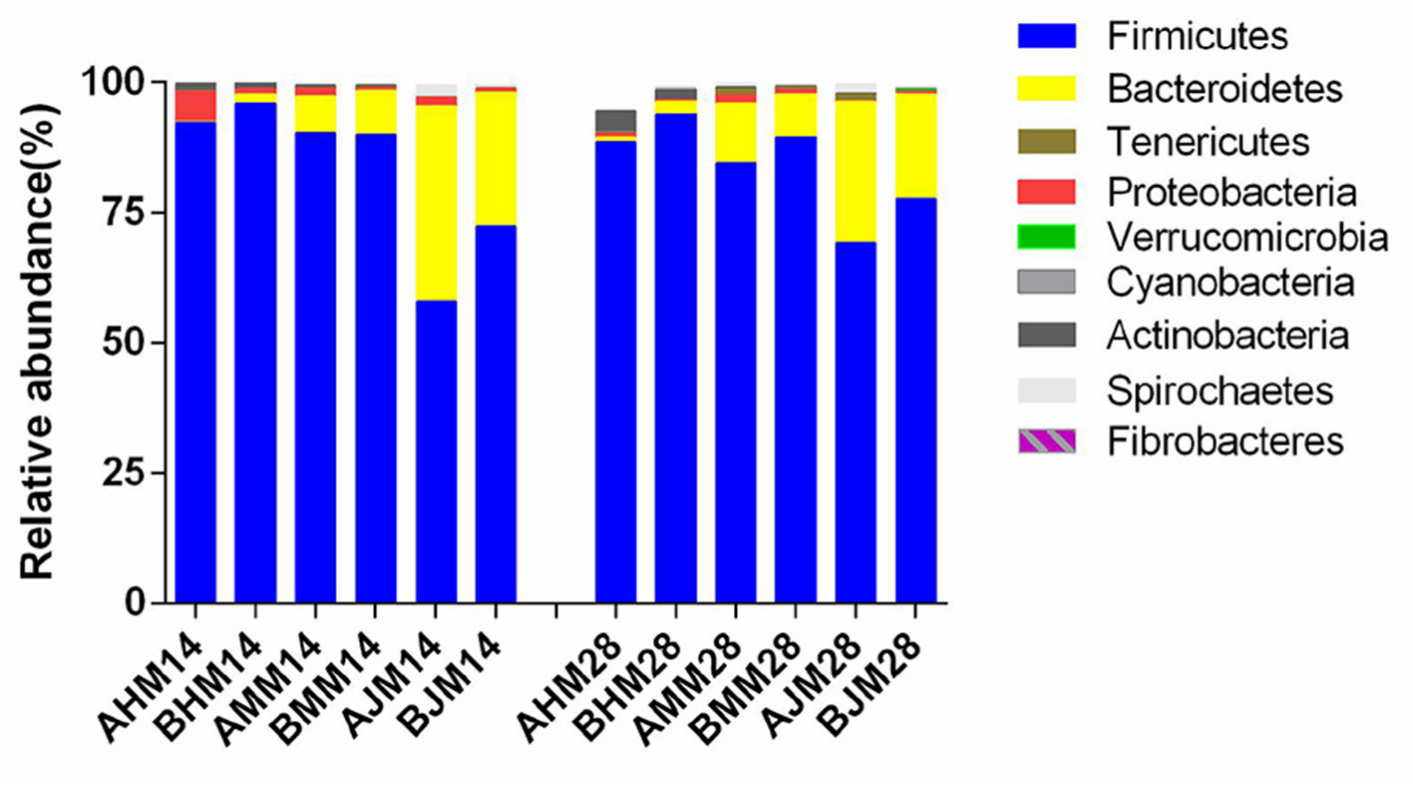
Effects of *Lactobacillus delbrueckii* on the bacterial community at the phylum level in the guts of the weaned piglets. AHM, AMM, and AJM represent ileum, cecum, and colon samples of the CON group, respectively; BHM, BMM, and BJM represent ileum, cecum, and colon samples of the LAC group, respectively; 14 and 28 represent the 14 and 28 days of the trial period, respectively.

The microbial compositions in the ileum, cecum, and colon of the weaned piglets are shown at the phylum level in Figure 4. The two main bacteria phyla were Firmicutes and Bacteroidetes in the ileum, cecum, and colon, with Actinobacteria, Proteobacteria, Cyanobacteria, and Tenericutes also presented in the three sections. The relative abundances of the different phylum in the gut are shown in Table 5. There were no significant differences in the relative abundances of bacterial phylum in the ileum between the LAC group and the CON group throughout the entire experimental period (*p* > 0.05). On the 28th day, piglets in the LAC group exhibited higher *Firmicutes* content in the cecum compared with the piglets in the CON group (*p* < 0.05). On the 14th day, the piglets in the LAC group exhibited higher relative abundance of Firmicutes but lower relative abundance of Bacteroidetes in the cecum compared with the CON group (*p* < 0.05). However, on the 28th day, the piglets in the LAC group exhibited higher relative abundances of Firmicutes and Fibrobacteres, but lower relative abundances of Bacteroidetes and Actinobacteria in the cecum compared with the piglets in the CON group (*p* < 0.05).

**Table 5:** Effects of *Lactobacillus delbrueckii* on some bacterial phyla in the weaned piglets.

**Gut**	**Item**	**Time**	**CON**	**LAC**	**SEM**	***P*-value**
Cecum	*Firmicutes*	28 days	84.59^b^	89.52^a^	1.89	0.029
	*Spirochaetes*	28 days	0.71^a^	0.37^b^	0.18	0.049
Colon	*Bacteroidetes*	14 days	37.45^a^	25.72^b^	5.81	0.032
	*Firmicutes*	14 days	57.98^b^	72.34^a^	7.43	0.022
	*Actinobacteria*	28 days	0.25^a^	0.20^b^	0.01	0.012
	*Bacteroidetes*	28 days	27.09^a^	20.14^b^	2.19	0.05
	*Fibrobacteres*	28 days	0.01^b^	0.03^a^	0.01	0.032
	*Firmicutes*	28 days	69.14^b^	77.70^a^	2.51	0.022

*CON, basal diet; LAC, basal diet group with 0.1% Lactobacillus delbrueckii. Microbiota taxa composition at the phylum levels by relative abundance is shown. Different superscript lowercase letters within each group mean different significantly (p < 0.05). Each mean represents four replicates*.

**Figure 5:**
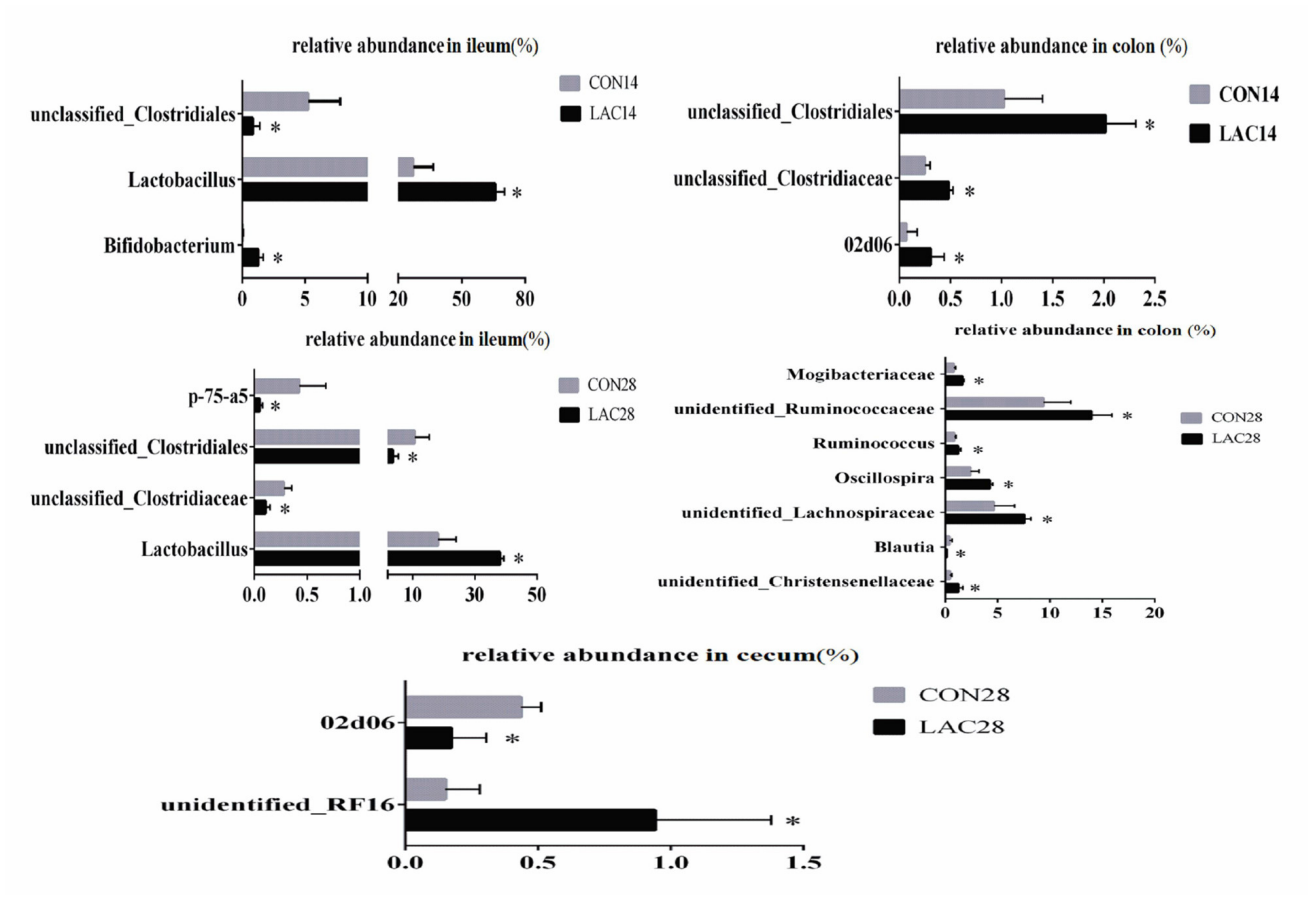
Effects of *Lactobacillus delbrueckii* on the intestinal bacterial community at the genus level in the weaned piglets. CON, basal diet; LAC, basal diet group with 0.1% *Lactobacillus delbrueckii*; 14 and 28 represent the 14 and 28 days of the trial period, respectively. *N* = 4. Label (*) mean significant difference between the CON group and the LAC group (*p* < 0.05).

Changes in the bacterial communities at the genus level were noticed in the ileum, cecum, and colon of weaned piglets between the two groups, which are shown in Figure 5. On the 14th day, piglets in the LAC group exhibited higher relative abundance of *Bifidobacterium* and *Lactobacillus*, but lower relative abundance of unclassified_*Clostridiales* in the ileum (*p* < 0.05). Moreover, piglets in the LAC group exhibited higher relative abundance of 02d06 and unclassified *Clostridiales* in the colon (*p* < 0.05). At the 28th day, piglets in the LAC group exhibited higher relative abundance of *Lactobacillus*, but lower relative abundance of unclassified_*Clostridiales* and p-75-a5 in the ileum (*p* < 0.05). Additionally, piglets in the LAC group exhibited higher relative abundance of unidentified_*RF16*, but lower relative abundance of 02d06 in the cecum on the 28th day (*p* < 0.05). Finally, the piglets in the LAC group exhibited higher relative abundance of unidentified *Christensenellaceae*, unidentified *Lachnospiraceae, Ruminococcus*, unidentified *Mogibacteriaceae, Oscillospira*, unidentified *Ruminococcaceae*, but lower relative abundance of *Blautia* in the colon on the 28th day (*p* < 0.05).

### Short-Chain Fatty Acid Quantification

**Figure 6:**
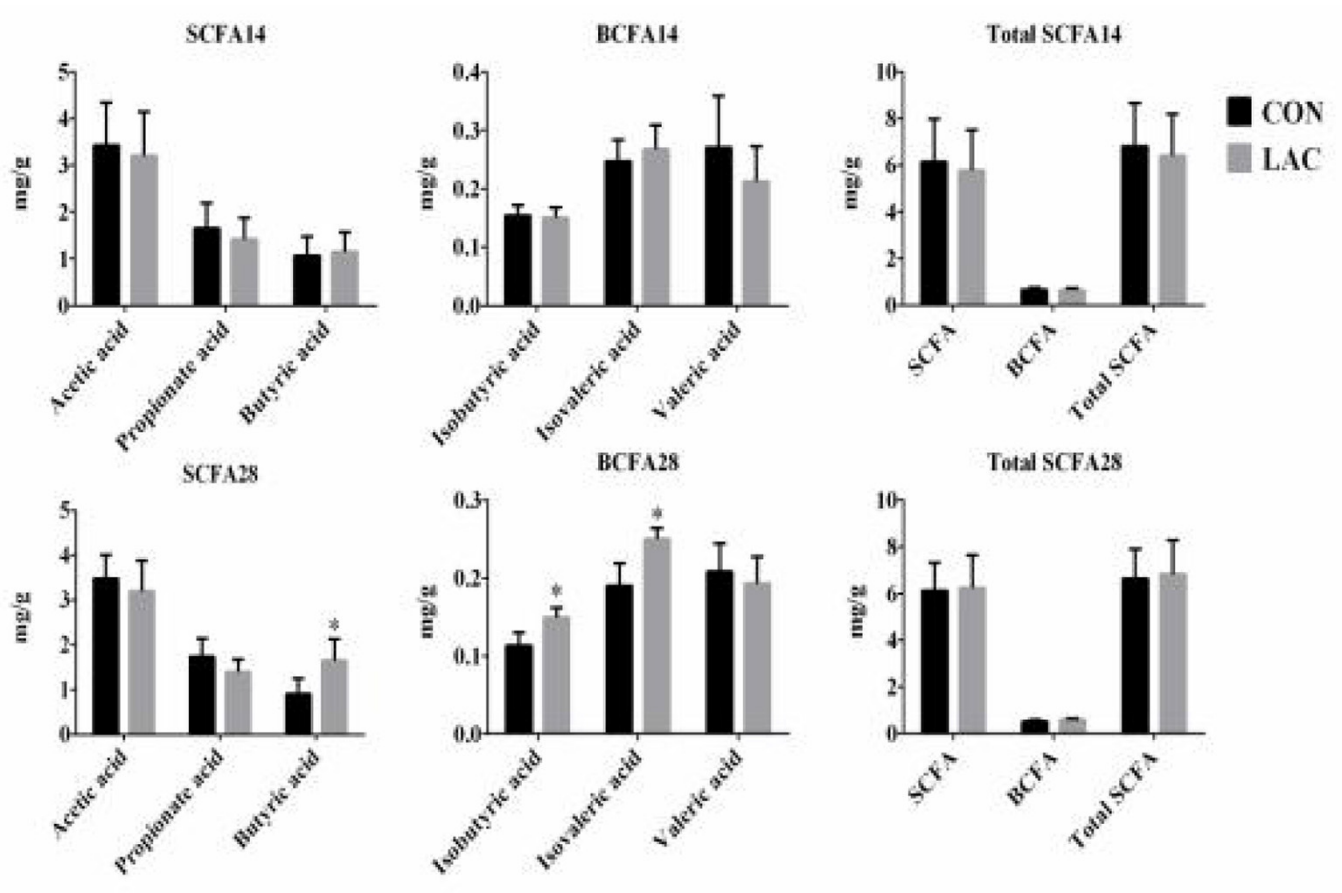
Effects of *Lactobacillus delbrueckii* on the concentrations of the short-chain fatty acids in the colon digesta of the weaned piglets. CON, basal diet; LAC, basal diet group with 0.1% *Lactobacillus delbrueckii*; SCFA, short-chain fatty acid, including acetic acid, propionic acid, and butyric acid BCFA, branched-chain fatty acid, including isobutyric acid, isovaleric acid, and valeric acid. Total SCFA, total short-chain fatty acid, including acetic acid, propionic acid, butyric acid, isobutyric acid, isovaleric acid, and valeric acid. N = 4. Label (*) mean significant difference between the CON group and the LAC group (*p* < 0.05); 14 and 28 represent the 14 and 28 days of the trial period, respectively.

The SCFA concentrations in the colonic chyme are shown in Figure 6. No significant differences were observed in the total SCFA concentration in the colonic chyme between the LAC group and the CON group on the 14th day (*p* > 0.05). On the 28th day, the concentrations of butyrate, isobutyric acid, and isovaleric acid in the colonic chyme increased significantly in the LAC group (*p* < 0.05). However, there were no differences in the concentrations of acetate, propionate, valeric acid, and total SCFA in the LAC group compared with the CON group.

## Discussion

Probiotics have beneficial effects on the host by enhancing the growth performance and providing the immunological protection (19). *Lactobacillus* species are one taxon of the resident bacteria in the gastrointestinal tract of most animals, which are usually used as probiotics (20, 21). The results of the present study showed that dietary supplementation of *Lactobacillus delbrueckii* enhanced the ADG of the weaned piglets, which was consistent with the results of previous research that oral administration of *Lactobacillus delhrueckii* during the suckling period increased the ADG of pre-weaning piglets (11). One possible reason for the result could be the improvement in nutrient absorption. The phyla Firmicutes and Bacteroidetes were known for polysaccharide fermentation. It was reported that when the ratio of Firmicutes/Bacteroidetes improved, the host was able to absorb more energy from the diet, and the ability of energy storage was strengthened (22). Our results showed that *Lactobacillus delhrueckii* significantly increased the relative abundance of Firmicutes, but decreased the relative abundance of Bacteroidetes in the colon of the piglets. Additionally, there were more beneficial bacteria and less potential pathogenic bacteria in the *Lactobacillus delhrueckii*-treated group, which might contribute to the higher daily weight gain (22). In contrast, it was reported that dietary supplementation of multiple *Lactobacillus (containing six strains of the genus Lactobacillus)* has no effect on the daily weight gains of the weaning pigs (23). Such variable results might probably be caused by the variations in the genetic background, health condition, diet composition, and feeding method of the piglets.

The present study showed that dietary supplementation of *Lactobacillus delhrueckii* had limited effects on the serum biochemical parameters. Basal diet with 0.1% *Lactobacillus delbrueckii* did not affect the serum levels of AST, LDL, TC, and TG. These data indicated no negative actions of *L. delbruecki* on the lipid metabolism of the piglets. The HDL concentration of piglets in the LAC groups was higher on the 28th day than the CON group, indicating that supplementation of *Lactobacillus delhrueckii* enhanced the lipid utilization on the 28th day of the trial period. On the other hand, the TP concentration of piglets in the LAC group was higher on the 14th day. Changes in the serum level of TP reflected the utilization efficiency of proteins, that the serum total protein could be inhibited when the feed intake decreased (24). Our result indicated that supplementation of *Lactobacillus delhrueckii* could improve ADFI, representing the enhancement of the protein synthesis ability. The increased serum concentration of TP was suggested to be an indicator of enhanced immune capability, which was considered to be direct reference to the body's immune function (25). *Lactobacillus delbrueckii* did not affect the serum levels of AST and ALT of the piglets in the LAC group on the 28th day. AST and ALT were principally found in the liver and were considered to be biomarkers for liver cell damages (26). The lower ALT values in the piglets treated with *Lactobacillus delhrueckii* indicated that *Lactobacillus delhrueckii* might have protective effects on the liver functions. It was consistent with the previous result that *Lactobacillus* supplementation decreased the serum ALT levels in lactating sows (27). The effects of *Lactobacillus delhrueckii* on serum parameters might be related to the supplemented rate of *Lactobacillus delhrueckii* and the time range of the experiment.

In this study, we found that dietary supplementation of *Lactobacillus delhrueckii* had little effects on the intestinal mucosal morphology of piglets on the 14th day, but increased the villus height and V/C value in the ileum on the 28th day. A reduced number of proteobacteria and pH of the small intestine could improve the morphology of intestinal mucosa (28, 29). Increasing villus height and V/C value were widely recognized to improve the growth performance of piglets. The supplement of probiotics to weaning piglets and broilers caused higher villi in the small intestine and higher proliferation of the endothelial cell (30). However, the villus length, crypt depth, and goblet cell number in the small intestine of the weaned pig were not affected by the probiotic treatments (31). Such variable results might be attributed to the variations in the types of the probiotics, animals, and experiment times. Similarly, previous research demonstrated that oral administration of *Lactobacillus delbrueckii* during the suckling period improved the villus height (on day 21) and the V/C ratio (on days 21 and 28) of the jejunum in piglets after weaning (11). Hence, our results indicate that dietary supplement of *Lactobacillus delbrueckii* plays a beneficial role in improving the intestinal morphology and pH of the weaned piglets.

As observed in the present study, dietary *Lactobacillus delhrueckii* had significant effects on the Chao 1 and Shannon of alpha diversity indexes of the bacteria in the ileum, cecum, and colon. This result suggested that the diversity of microbial community changed with *Lactobacillus delhrueckii* supplementation. Dietary *Lactobacillus delbrueckii* had no effect on the phylum's abundance in the ileum of the weaned piglets. Basal diet with 0.1% *Lactobacillus delbrueckii* significantly increased the relative abundance of Firmicutes but decreased the relative abundance of Bacteroidetes in the colon. Taxonomic profiling data demonstrated that LAC increased the level of Firmicutes on the cecum and colon, but reduced the level of Actinobacteria on the colon. Supplementation of *Lactobacillus*-based fermentation product (1 × 10^11^ CFU *Lactobacillus* casei/kg) in the diet of weaned piglets was reported to reduce the population of *E. coli* in the cecum and *Salmonella* spp. in the ileum (32). Additionally, it was reported that the relative abundance of Firmicutes increased, and the relative abundance of Bacteroidetes decreased with an increasing body mass index (BMI) in humans (33). The dietary supplementation of *Lactobacillus delbrueckii* could increase the ADG of the weaned piglets. A possible explanation was that Firmicutes could promote carbohydrate absorption more effectively compared with the Bacteroidetes, therefore, causing more weight gain of the host (34, 35). The analysis of the bacterial community at the genus level in the ileum revealed that *Lactobacillus delbrueckii* significantly increased the relative abundance of the genus of *Bifidobacterium* and *Lactobacillus*, but decreased the relative abundance of the genus of unclassified_*Clostridiales*. It was reported that *Lactobacillus* was one of the main bacterial groups found in the gastrointestinal tract (36). The genus *Bifidobacteria* existed in the gastrointestinal tract in both animals and humans, and it helped to maintain the balance of microorganisms in the gastrointestinal tract by reducing the pathogenic microbes. Therefore, *bifidobacteria* was associated with good health status of the host (37, 38). Similarly, previous researches indicated that the addition of *Lactobacilli* isolated from gastrointestinal tract in piglets (*Lactobacillus gasseri, L. reuteri, L. acidophilus, L. fermentum, L. johnsonii*, and *Lactobacillus mucosae*) increased the numbers of *lactobacilli* and *Bifidobacterium*, and reduced the numbers of *E. coli* and aerobic bacteria in the jejunum, ileum, cecum, and colon mucosa (37, 39). The present experiment showed that the weaned piglets receiving the *Lactobacillus delbrueckii* diet had increased number of *unidentified_RF16, Ruminococcus*, unidentified_*Ruminococcaceae*, unidentified_*lachnospiraceae*, and unidentified *Mogibacteriaceae*, which were SCFA producers (40, 41). These results indicated that *Lactobacillus delbrueckii* could robust a more symbiotic intestinal microflora, which was a benefit to the host.

To further elucidate the underlying mechanisms of the administered *Lactobacillus delbrueckii* altering the gut health in piglets, the intestinal pH and the concentrations of SCFA in the colon were analyzed. *Lactobacillus* species could tolerate oxygen for short periods and alter the gut microbiota composition and the SCFA productions (42). As observed in the current study, dietary *Lactobacillus delbrueckii* significantly increased the relative abundance of the butyric acid, iso-butyric acid, and iso-valeric acid at the colon in phase II. The acetate produced by gut bacteria helped in reducing the permeability of the intestinal mucosal (39). In this study, the weaned piglets fed with a diet containing *Lactobacillus delbrueckii* had lower digesta pH in the duodenum and ileum, with a tendency toward a lower jejunum pH in phase II. In general, all *Lactobacillus* species could reduce pH by producing lactic acid as the final product in the carbohydrate fermentation, thus, inhibiting the colonization of the pathogenic bacteria (43). This finding was confirmed by our data showing that application of *Lactobacillus delbrueckii* produced higher SCFA in the colon and lowered the digesta pH in the duodenum and ileum of the piglets in phase II. Therefore, we concluded that *Lactobacillus delbrueckii* could improve the intestinal health of the piglets.

## Conclusion

The present study demonstrates that dietary supplementation of 0.1% *Lactobacillus delbrueckii* could improve the mucosal morphology and cecal microflora of the weaned piglets, thus, improving the growth performance of the weaned piglets. These results indicate that *Lactobacillus delbrueckii* could be used as a potential feed supplement for weaned piglets.

## Data Availability Statement

The original contributions presented in the study are included in the article/Supplementary Material, further inquiries can be directed to the corresponding author/s.

## Ethics Statement

The animal study was reviewed and approved by All the experimental procedures were approved by the Institutional Animal Care and Use Committee of Hunan Agricultural University (Changsha, China) (No. 2017-09).

## Author Contributions

X-LW and X-GH conceptualized the study and performed the validation. X-LW, Y-LL, and Y-HL formulated the methodology. L-YY provided the software. JY, X-LW, and Z-YL performed the formal analysis. Data curation was done by X-LW and Z-YL. X-LW wrote the original draft. D-XH, Z-YL, and J-HH reviewed and edited the manuscript. All authors contributed to the article and approved the submitted version.

## Conflict of Interest

Y-LL was employed by the company Hunan Pufeike Biotechnology. The remaining authors declare that the research was conducted in the absence of any commercial or financial relationships that could be construed as a potential conflict of interest.

## Publisher's Note

All claims expressed in this article are solely those of the authors and do not necessarily represent those of their affiliated organizations, or those of the publisher, the editors and the reviewers. Any product that may be evaluated in this article, or claim that may be made by its manufacturer, is not guaranteed or endorsed by the publisher.
