# Supplementary material for: Effects of Dietary Supplementation of Lactobacillus delbrueckii on Gut Microbiome and Intestinal Morphology in Weaned Piglets
Source: Front Vet Sci. 2021 Aug 19;8:692389. doi: 10.3389/fvets.2021.692389 (PMC8417114; doi:10.3389/fvets.2021.692389)
Supplement: Supplementary file 1 [file Table_1.DOC]

**Table S1.** Different intestinal genera of taxa in weaned piglets by supplement of *L. delbrueckii*

| Time(d) | Gut | Phylum | Order | Family | Genus |  |
| --- | --- | --- | --- | --- | --- | --- |
| 14^th^d | Ileum | Actinobacteria | Bifidobacteriales | Bifidobacteriaceae | Bifidobacterium |  |
|  | Ileum | Firmicutes | Lactobacillales | Lactobacillaceae | Lactobacillus |  |
|  | Ileum | Firmicutes | Clostridiales | unclassified_Clostridiales | unclassified_Clostridiales |  |
|  | colon | Firmicutes | Clostridiales | Clostridiaceae | 02d06 |  |
|  | colon | Firmicutes | Clostridiales | Clostridiaceae | unclassified_Clostridiaceae |  |
|  | colon | Firmicutes | Clostridiales | unclassified_Clostridiales | unclassified_Clostridiales |  |
| 28^th^d |  |  |  |  |  |  |
|  | Ileum | Firmicutes | Lactobacillales | Lactobacillaceae | Lactobacillus |  |
|  | Ileum | Firmicutes | Clostridiales | Clostridiaceae | unclassified_Clostridiaceae |  |
|  | Ileum | Firmicutes | Clostridiales | unclassified_Clostridiales | unclassified_Clostridiales |  |
|  | cecum | Firmicutes | Erysipelotrichales | Erysipelotrichaceae | p-75-a5 |  |
|  | cecum | Bacteroidetes | Bacteroidales | RF16 | unidentified_RF16 |  |
|  | cecum | Firmicutes | Clostridiales | Clostridiaceae | 02d06 |  |
|  | colon | Firmicutes | Clostridiales | Christensenellaceae | unidentified_Christensenellaceae |  |
|  | colon | Firmicutes | Clostridiales | Lachnospiraceae | unidentified_Lachnospiraceae |  |
|  | colon | Firmicutes | Clostridiales | Ruminococcaceae | Oscillospira |  |
|  | colon | Firmicutes | Clostridiales | Ruminococcaceae | Ruminococcus | |
|  | colon | Firmicutes | Clostridiales | Ruminococcaceae | unidentified_Ruminococcaceae | |
|  | colon | Firmicutes | Clostridiales | Mogibacteriaceae | Unidentified Mogibacteriaceae | |
|  | colon | Firmicutes | Clostridiales | Lachnospiraceae | Blautia | |

Note. 14^th^d: on the 14^th^ day in the experimental time, 28^th^d: on the 28^th^ day in the experimental time.
